# Flavor Classification/Categorization and Differential Toxicity of Oral Nicotine Pouches (ONPs) in Oral Gingival Epithelial Cells and Bronchial Epithelial Cells

**DOI:** 10.3390/toxics10110660

**Published:** 2022-10-31

**Authors:** Sadiya Bi Shaikh, Wai Cheung Tung, Cortney Pang, Joseph Lucas, Dongmei Li, Irfan Rahman

**Affiliations:** 1 Department of Environmental Medicine, School of Medicine and Dentistry, University of Rochester Medical Center, Rochester, NY 14642, USA; 2 Department of Clinical and Translational Science Institute, School of Medicine and Dentistry, University of Rochester, Rochester, NY 14642, USA

**Keywords:** oral nicotine pouches, inflammation, cytotoxicity, periodontal problems, pulmonary health

## Abstract

Oral nicotine pouches (ONPs) are a modern form of smokeless tobacco products sold by several brands in the U.S., which comprise a significant portion of non-combustible nicotine-containing product (NCNP) sales to date. ONPs are available in various flavors and may contain either tobacco-derived nicotine (TDN) or tobacco-free nicotine (TFN). The growth in popularity of these products has raised concerns that flavored ONPs may cause adverse oral health effects and promote systemic toxic effects due to nicotine and other ONP by-products being absorbed into the circulatory system through oral mucosa. We hypothesized that flavored ONPs are unsafe and likely to cause oral and pulmonary inflammation in oral and respiratory epithelial cells. Before analyzing the effects of ONPs, we first classified ONPs sold in the U.S. based on their flavor and the flavor category to which they belonged using a wheel diagram. Human gingival epithelial cells (HGEP) were treated with flavored ONP extracts of tobacco (original, smooth), menthol (wintergreen and cool cider), and fruit flavor (americana and citrus), each from the TDN and TFN groups. The levels of ONP-induced inflammatory cytokine release (TNF-α, IL-6, and IL-8) by ELISA, cellular reactive oxygen species (ROS) production by CellRox Green, and cytotoxicity by lactate dehydrogenase (LDH) release assay in HGEP cells were assessed. Flavored ONP extracts elicited differential toxicities in a dose- and extract-dependent manner in HGEP cells 24 h post-treatment. Both fruit TDN and TFN extracts resulted in the greatest cytotoxicity. Tobacco- and fruit-flavored, but not menthol-flavored, ONPs resulted in increased ROS production 4 h post-treatment. Flavored ONPs led to differential cytokine release (TNF-α, IL-6, and IL-8) which varied by flavor (menthol, tobacco, or fruit) and nicotine (TDN vs. TFN) 24 h post-treatment. Menthol-flavored ONPs led to the most significant TNF-α release; fruit TFN resulted in the most significant IL-6 release; and fruit TDN and tobacco TFN led to the highest release of IL-8. Subsequently, human bronchial epithelial cells (16-HBE and BEAS-2B) were also treated with flavored ONP extracts, and similar assays were evaluated. Here, the lowest concentration treatments displayed increased cytotoxicity. The most striking response was observed among cells treated with spearmint and tobacco flavored ONPs. Our data suggest that flavored ONPs are unsafe and likely to cause systemic and local toxicological responses during chronic usage.

## 1. Introduction

**Figure 1:**
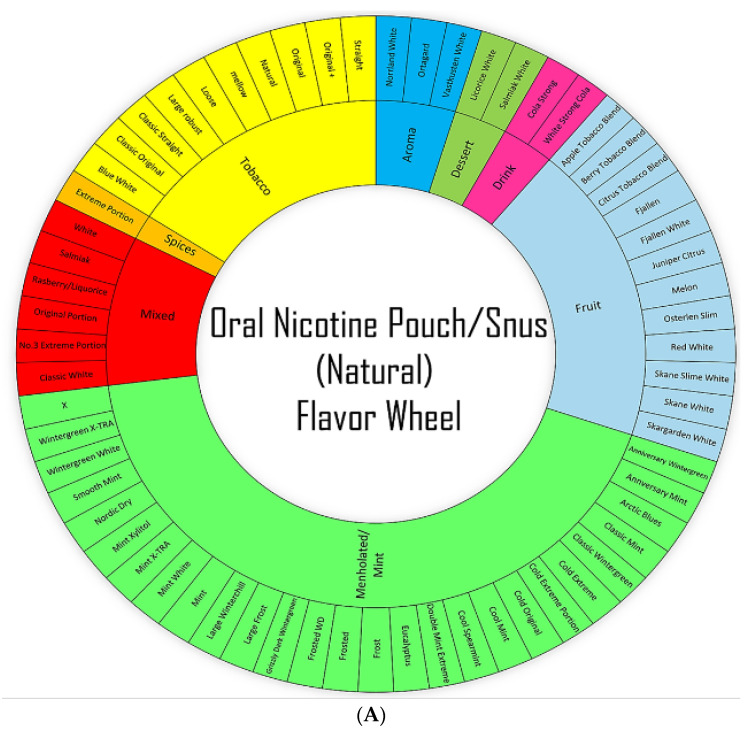

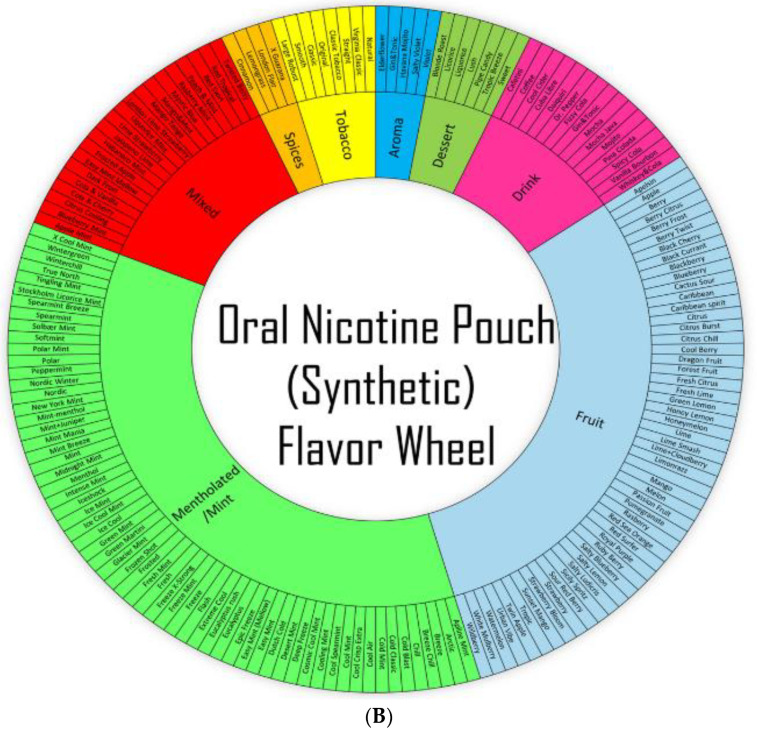
**Wheel-based Classification/Categorization of Natural/Synthetic Oral Nicotine Pouches (ONPs)/products commonly sold in the U.S**. (**A**) Flavor wheel for Natural/Snus Oral Nicotine Pouch (**B**) Flavor wheel for Synthetic oral Nicotine Pouch. The nicotine concentration of all smoke-free nicotine-based pouches ranges from 3 mg to 8 mg per pouch; mint/menthol and fruit are two of the most widely sold flavors in the U.S. The flavors of each pouch product in the diagram are color-coded by flavor category, Bright green Color represents ONPs of mint/menthol flavors, Light blue for fruit flavors, Red color mixed flavors, Pink color ONPs available in drink flavors, Yellow color for Tobacco flavors ONPs, parrot green color ONPs available in dessert flavors, Sky blue ONPs in aroma flavors and Orange represents ONPs available in flavor of spices. The inner wheel represents the most common flavors, and the outer wheel represents specific flavors.

**Figure 2:**
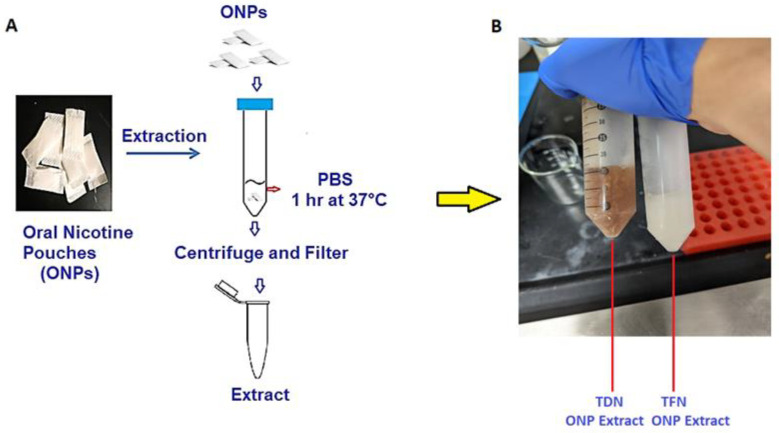
**Protocol for preparation of TDN (natural) and TFN (synthetic) nicotine pouches extract for cell exposures.** (**A**) Extracts of oral nicotine pouches were prepared by incubating pouches in PBS (1:10 *w*/*v*) for 1 h on a shaker (500 rpm) at 37 °C. Extracts were centrifuged and then filtered through 0.45 micron sterile filters and denoted 100% for treatments. (**B**) The appearance of freshly extracted TDN (natural) and TFN (synthetic) extracts are shown.

According to the 2021 National Youth Tobacco Survey (NYTS), approximately 2.55 million middle and high-school students in the United States (U.S.) reported their current usage of tobacco products: specifically, 2.06 million (13.4%) high school students and 470,000 (4.0%) middle school students [1]. The prevalence of tobacco-product usage among youths in the U.S. is due to the emergence of non-combustible nicotine-containing products (NCNPs), such as electronic nicotine delivery systems (ENDS) or e-cigarettes, smokeless tobacco, and nicotine pouches [1]. Most alternative tobacco products sold in the U.S. still contain nicotine, which, in addition to being highly addictive, is known to cause injurious responses in the lungs, heart, and kidneys [2]. Youth studies have shown that nicotine exposure in adolescence induces lasting effects into adulthood, including emotional dysregulation and decreased cognitive functioning [3]. Due to its popularity among students, oral nicotine pouches (ONPs) are an NCNP of growing concern; among students surveyed in the 2021 NYTS, 17.2% had frequently used ONPs [1]. Like Snus (a smokeless tobacco product), ONPs are pouch-based nicotine products—products relying on the absorption of nicotine into the oral mucosa [4]. Unlike Snus, however, ONPs contain no components of the tobacco plant’s leaves, stem, or dust. Some ONPs may contain tobacco-derived nicotine (TDN) though they lack any other components of the tobacco plant [5]. ONPs can come in various flavors (mint, fruity, tobacco, citrus, coffee, wintergreen, and berry), as represented in Figure 1A,B. Moreover, the availability of this multitude of flavors amongst ONPs contributes to the prevalence of ONP usage in the U.S. [6]. We have recently identified, via online discussion forums and social media posts, increasingly positive attitudes towards ONPs among the online news forums/topic-discussion threads focusing on the usage of NCNPs [7]. Social media platforms such as social news websites and forum boards—boards/platforms where users of ONPs and other NCNPs can actively share and discuss their experiences of different products, are a significant factor in influencing attitudes and consumer behaviors revolving around ONPs [7]. ONPs were prepared in Phosphate Buffer Saline (PBS) as shown in Figure 2A,B. 

On 15 March 2016, the FDA finalized the “Deeming Tobacco Products to Be Subject to the Federal Food, Drug, and Cosmetic Act” (the “Deeming Rule”) [8]. Under the deeming rule, the FDA’s regulatory authority over tobacco products was extended to all products containing TDN, including ONPs [8]. Subsequently, manufacturers of ONPs containing TDN were subject to pre-market assessment and were required to submit specific product information to the FDA as well as to comply with marketing restrictions [9]. In the past, ONPs may have contained synthetic nicotine instead of TDN. However, as of 14 April 2022, the FDA’s regulatory authority was extended to include ONPs utilizing synthetic nicotine or tobacco-free nicotine (TFN) [10]. On 15 March 2022, the definition of a “tobacco product” under the Federal Food, Drug, and Cosmetic Act was amended, as a result, to include “any product made or derived from tobacco or containing nicotine from any source, that is intended for human consumption” [10]. Now, any ONP, regardless of whether it uses TFN or TDN, which has not submitted a pre-market application (PMTA) to the FDA, will be removed from the market in the U.S. [8,10]. In terms of marketing, nicotine pouch sales increased from 163,178 units (USD 709,635) in 2016 to 45,965,455 units (USD 216,886,819) by the end of June 2020 in the U.S. [11]. Moreover, from 2020 to 2021, shipments of nicotine pouches to the U.S. from Zyn (manufactured in Sweden) increased by more than 50% [12].

Despite the significant increase in the of ONPs in the U.S., limited studies have been conducted so far that have delved into understanding the systemic-oral-pulmonary health risks of regular usage of ONPs or Snus [4,12,13,14]. However, studies have shown that regular usage of smoke-free nicotine pouch-based products of smokeless tobacco is associated with a higher risk for Parkinson’s disease, cancer, birth defects, type 2 diabetes, oral submucosal fibrosis, of cardiovascular disease [15,16]. Regarding studies on the health effects of Snus and ONPs, limited studies and case reports have focused on investigating the systemic-oral-pulmonary health risks of regularly using these products [17,18,19,20,21]. While smokeless nicotine-based products, including Snus and ONPs, are not inhaled through the lungs, the nicotine, flavoring chemicals, and by-products within those pouches can be absorbed across the buccal membrane into the systemic circulation. These by-products can act locally on other tissues within the body, which may affect the cardiopulmonary system via the microvasculature, liver, kidneys, the pancreas, and the esophagus [18,19,20,21,22,23,24]. There is a potential for these byproducts absorbed from Snus and ONPs to interact with the airways and lung microvasculature. Regarding other ways oral pouches/smokeless tobacco has been shown to impact the lungs: previous case studies have reported that pulmonary aspirations of smokeless tobacco products induced multifocal airway obstructions and recurrent pulmonary infiltrations in the lungs of patients, likely resulting in aspiration pneumonia [19,24]. Smokeless tobacco-induced aspiration pneumonia and subsequent pulmonary inflammation can potentially be caused by direct contact between the airways and saliva that has come into contact with smokeless tobacco. Oral submucosal fibrosis is likely associated with pulmonary complications as seen with chewing tobacco. Studies have shown that smokeless tobacco usage significantly reduces antioxidant activity in saliva and significantly increases the level of toxic metals in saliva, including heavy metals known to induce the formation of reactive oxygen species (ROS) in lung epithelial cells [25,26]. Additionally, smokeless tobacco-induced pulmonary inflammation may also be caused by regurgitated gastric stomach acid coming into contact with the airways; this occurs because nicotine is absorbed into the bloodstream from oral pouches/smokeless tobacco increasing the risks of lower esophageal sphincter relaxation [27,28].

Due to the potential of smokeless nicotine pouch products negatively impacting lung function, the limited number of relevant studies, and the increasing popularity of ONPs in the U.S., we have conducted a study involving the analysis of changes in inflammatory cytokine release, ROS production, and cytotoxicity in bronchial/lung epithelial cells exposed to smoke-free nicotine-based pouch extract. Unlike previous studies conducted, which include analyses of cytotoxicity and cellular ROS among bronchial epithelial cells exposed to a variety of flavored ONPs, our study includes analyses using four of the most widely sold brands of ONPs containing TFN in the U.S., as well as analyses of inflammatory cytokine levels among bronchial epithelial cells exposed to these ONPs.

In the current study, we aimed to investigate the cytotoxicity and inflammatory profile of the ONPs of both TDN and TFN categories among tobacco, menthol, and fruit flavored groups. For our first objective, it was important to understand what was commercially available on the market. We classified and categorized the brands and flavors of both TDN and TFN oral nicotine products available via a wheel-based system. We next sought to examine the effect of these ONPs on dental and pulmonary health. For studying this, human gingival epithelial cells (HGEP) cells were employed and treated with one of six flavored ONPs belonging to TDN or TFN groups. We further analyzed cellular cytotoxicity; cellular ROS; and cytokine release of inflammatory cytokines such as tumor necrosis factor–α (TNF-α), interleukin-6 (IL-6), and interleukin-8 (IL-8) post ONP treatment. Similarly, human bronchial epithelial cells such as BEAS-2B and 16-HBE cell lines were also used in this study and were treated with five ONPs. To best of our knowledge, our study is the first to attempt to classify/categorize and to elucidate how the usage of ONPs may impact both the oral and lung epithelium.

## 2. Materials and Methods

### 2.1. Classification/Categorization of Oral Nicotine Pouches (ONPs)/Products

The pouches were procured from local vendors based in Rochester, NY, USA, and are sold publicly with age restrictions. The classification/categorization of the extracts of different flavored ONPs was carried out based on natural or synthetic flavor categorizations and nicotine concentrations (Table 1 and Table 2 and Figure 1A,B). The nicotine pouches are sold at different strengths, ranging from 3 mg to ~8 mg/pouch, and contain various levels of moisture content and alkalinity. ONPs generally contain sweeteners, flavorings, food grain fillers, and plant-based fibers (cellulose). The flavors and flavor categories of ONPs belonging to tobacco derived/tobacco free categories and to the most widely sold brands in the U.S are drawn in the form of a wheel diagrams (Figure 1A,B). The flavors of ONPs made by Rogue (Swisher) include wintergreen, peppermint, spearmint, berry, apple, honey lemon, mango, and cinnamon. ONP flavors from ON! include wintergreen, cinnamon, citrus, coffee, berry, and original. ONP flavors from Velo, formerly REVEL brand, include berry, cherry, cinnamon, citrus, coffee, dragon fruit, mint, wintergreen, peppermint, cream, vanilla, and spearmint. ONP flavors from Zyn (Swedish Match) include coffee, cinnamon, wintergreen, spearmint, citrus, peppermint, cool mint, and original/smooth tobacco (unflavored or tobacco flavored). The NIIN ONP flavors include wintergreen, spearmint, cool mint, citrus chill, and cinnamon. The FRE ONP flavors include sweet, lush, wintergreen, and mint. The Killa ONP flavors include cold mint, spearmint, Dutch cold, watermelon, blueberry, and apple. The Nordic Spirit ONPs include mint, spearmint, wild berry, mocha, and elderflower; ONP flavors from Zonex include cold blast (mint and peppermint), berry, and breeze. The flavors of Lyft ONPs include ice cool, mint, freeze X-strong, cool air, blueberry, lime, berry twist, blonde roast, melon, strawberry, licorice, and tropic. ONP flavors from Dryft (Kretek) include blackberry, cinnamon, citrus, coffee, dragon fruit, peppermint, spearmint, and wintergreen. Mint/mentholated flavors and fruit flavors of ONPS were analyzed in this study as these are two of the most widely sold ONP flavors in the U.S, according to one study using retail scanner data to assess nicotine pouch sales in the U.S from 2016 to 2020 [11].

### 2.2. Extraction of Oral Nicotine Pouches

As mentioned above, the extraction of the oral nicotine pouches was carried out in PBS is shown in Figure 2A,B. Extracts of oral nicotine pouches were created by incubating indicted pouches in PBS (1:10 *w*/*v*) for 1 h on a shaker (500 rpm) at 37 °C. Extracts were centrifuged and then filtered through 0.45-micron sterile filters and denoted 100% for treatments [29,30,31,32]. The ONP extracts were freshly prepared every time for experimental use and pH was evaluated to ensure it is alkaline in nature. Figure 2B depicts the color appearance after the extraction of ONPs. The brown color extracts were obtained after the extraction of TDN ONPs and TFN extracts were transparent in color.

### 2.3. Cells and Culture Conditions

#### 2.3.1. Human Gingival Cell Model to Study the Effect of ONP

Human gingival epithelial progenitors (HGEPp; gingival epithelial cells) were obtained from Accegen Biotechnology (Fairfield, USA). Cells were grown in Oral Epithelia Cell Culture Medium (ABM-TM4365), as recommended by Accegen Biotechnology and were maintained at 37 °C in a 5% CO_2_ humidified atmosphere. Prior to the experiments, cryopreserved HGEPp were recovered and cultured for a further 1–12 passages before disposal, and cells were further seeded and grown to 80% confluency before performing the assays.

#### 2.3.2. Human Bronchial Epithelial Cell Model to Study the Effect of ONP

The human bronchial epithelial cell line (16-HBE) and BEAS-2B cell line (ATCC) were used in this study. 16-HBE cells were cultured in Dulbecco’s Modified Eagle’s Medium (DMEM) supplemented with 10% FBS and 1% antibiotic-antimycotic solution. BEAS-2B cells were grown in DMEM/F12 media with 10% FBS, 15µM HEPES, and 1% antibiotic-antimycotic solution. Cells were maintained at 37 °C and 5% CO_2_ in a humidified atmosphere and used for the experiments. Passages below 10 were selected, and when the sufficient density was reached, cells were seeded at 250,000 cells per well in 48-well plates with 500 µL of complete DMEM media. Cells were incubated overnight in low serum-containing media (FBS 0%) to lessen undesired stimulation of the cells and the cytokine background levels. 

### 2.4. Treatment of Oral Nicotine Pouch Extracts to Oral and Bronchial Epithelial Cells and Collection of Conditioned Media

HGEPp cells were treated with six flavored ONPs, each categorized into TDN and TFN belonging to a tobacco/menthol/fruit flavors group of various brands (information provided in Table 1). After 24 h of ONP treatment, the conditioned medium was collected to measure the cytotoxicity and pro-inflammatory cytokines levels. The BEAS-2B/16 HBE cells were treated with five flavored ONPS as mentioned in Table 2. To minimize cell death when assessing for cellular ROS and cytokine release, 24 h post-treatment, the conditioned media was collected by centrifuging the HGEPp cell suspension at 879 rpm for 5 min and centrifuging the 16 HBE/BEAS-2B cell suspension at 1000 rpm for 5 min. Subsequently, collected culture media supernatants were frozen at −20 °C to assess cytokine levels.

The viability of both cell lines was measured by re-suspending the cells in their respective culture medium using the acridine orange (AO) and propidium iodide (PI) staining for evaluating the live and dead cell concentration as a percentage (for seeding) via an automatic cellometer. AOPI was purchased separately from Nexcelom Bioscience.

### 2.5. Lactate Dehydrogenase (LDH) Cytotoxicity Assay

Quantification of lactate dehydrogenase (LDH) release was used to assess the levels of cytotoxicity induced by exposure to extracts of ONPs and Snus. Respective treatments of nicotine pouches were used on the designated wells at varying concentrations in triplicates, for oral epithelial cells 0.25%, 0.05%, 1%, 3%, and 10% concentrations were analyzed whereas for bronchial epithelial cells 0.05%, 0.1%, 0.25%, and 1% concentrations in triplicates were applied [31,32]. Following treatment of the nicotine pouches, the culture medium was aspirated and centrifuged at 1000 rpm for 5 min to obtain a cell-free supernatant. The activity of LDH in the medium was determined using a commercially available kit (Roche) per the manufacturer’s instructions. Aliquots of medium and the required reagents were mixed in a 96-well plate, and absorbance was recorded at 490 nm using a microplate spectrophotometer system. The outcome was presented as a percentage of positive control (Triton X-100) values.

### 2.6. ROS Assay by CellROX Green

Both oral and bronchial epithelial cells were treated with TDN or TFN oral nicotine pouch extracts using the protocol described previously [33]. After 4 h of incubation, cells were stained with CellROX reagents (Green) and Hoechst 33342 was used as nuclear stain and images were viewed immediately. A broad spectrum of ROS was determined in living cells using the fluorogenic indicator CellROX. Probes for CellRox green were purchased from Invitrogen (Carlsbad, CA, USA) and applied according to the manufacturer’s instructions. Images were captured on a Cytation 5 cell imaging multimode reader (BioTek). Similar image acquiring times and settings for intensity were used for all images obtained. CellRox fluorescence were processed using Image J software and the corrected total cell fluorescence (CTCF) was evaluated using the formula: CTCF = (Integrated Density-Area of selected cell * Mean fluorescence of background readings).

### 2.7. Inflammatory Response (TNF-α, IL-8, and IL-6) Assay

After cells treatments, conditioned media was collected at different concentrations. Pro-inflammatory cytokine (TNF-α) release was determined using TNF-α ELISA kit (R&D systems), whereas (IL-8) and (IL-6) release was determined using the IL-6 and IL-8 ELISA kit (Invitrogen) according to the manufacturer’s instructions (ThermoFisher).

### 2.8. Statistical Analysis

Statistical analyses of significance were performed by the Student’s T-test or one-way ANOVA (Tukey’s/Dunnett’s multiple comparison tests) when comparing multiple groups using GraphPad Prism 7 (La Jolla, CA, USA). Data are presented as means ± SEM. *p* < 0.05 was considered statistically significant.

## 3. Results

### 3.1. Differential Cytotoxicity among Human Gingival Epithelium Progenitors (HGEPp) and Bronchial Epithelial Cells Exposed to Different Flavored Oral Smokeless Nicotine Products

#### 3.1.1. HGEPp Cells Were Treated with Various Flavored ONPs of Different Brands

**Figure 3:**
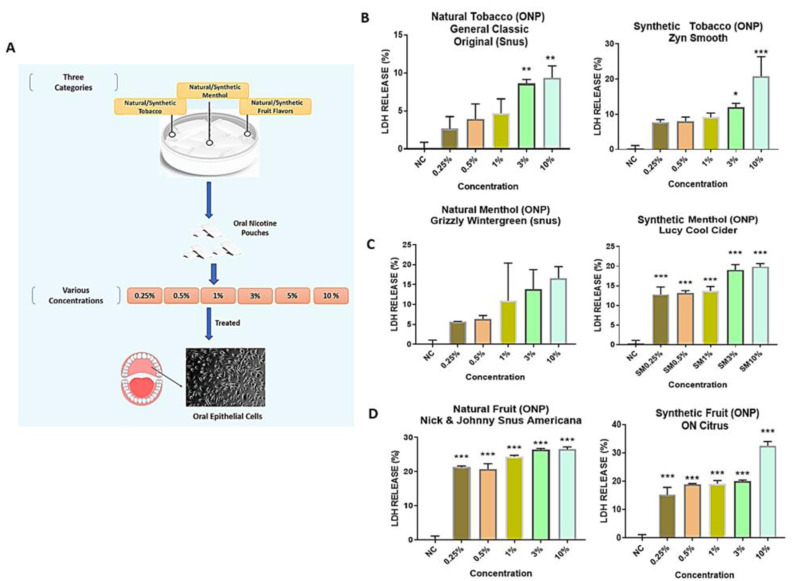
**Differential cytotoxicity among oral epithelial cells upon treatment with different flavored oral smokeless nicotine products**: (**A**) Diagrammatical representation of the experimental workflow. (**B**–**D**) Cytotoxicity Assay on oral epithelial cells: human gingival epithelium progenitors cells (HGEPp; gingival epithelial cells) were treated with different doses (0.25%, 0.5%, 1%, 3%, and 10%) of oral nicotine extracts categorizing natural/synthetic tobacco, menthol, and fruit flavors from different brands (Snus, Zyn, Grizzly, Lucy and ON). Followed by 24 h exposure, conditioned media were used for the lactate dehydrogenase (LDH) assay. Data represented as mean ± SEM, * *p* < 0.05, ** *p* < 0.01, *** *p* < 0.001 compared to control. *n* = 3.

To study the effect of ONPs on oral epithelial cells, each nicotine pouch was chosen belonging to tobacco, menthol, and fruit flavors (natural/synthetic nicotine groups). As mentioned above, ONPs extracts at 0.25%, 0.05%, 1%, 3% and 10% (*v*/*v*) concentrations were used to treat HGEPp cells as diagrammatically represented in Figure 3A. Furthermore, the cytotoxicity of ONP extracts were tested. Treatment with natural tobacco ONP belonging to the brand General (Snus) of classic original flavor at 3% and 10% (*v*/*v*) concentrations showed a significant increase in LDH (8–13%) release at 24 h post-treatment (** *p* < 0.01) as compared to the 0.25%, 0.5%, and 1% concentrations, which possessed less than 10% LDH release. Similar effects were observed with ZYN Smooth, which was a synthetic tobacco ONP, resulting in a significant increase in LDH (15–30% approx.) at 3% (* *p* < 0.05) and 10% (*** *p* < 0.001) doses (Figure 3B), whereas natural menthol pouches with wintergreen flavor of Grizzly brand depicted about 13–16% LDH release at 3% and 10% (*v*/*v*) doses, but these changes were not significant. Synthetic menthol Lucy Cool Cider demonstrated significantly increased (13–19%) LDH release at all the concentrations: 0.25%, 0.05%, 1%, 3%, and 10% (*v*/*v*) (*** *p* < 0.001) (Figure 3C), whereas the natural fruit citrus flavored ONP of Nick & Johnny Americana showed 21–27% of LDH release, and synthetic fruit flavored ONP of ON! indicated a 20–34% increase in LDH release, which was statistically significant (*** *p* < 0.001) at all applied concentrations (Figure 3D). Since both 3% and 10% (*v*/*v*) doses showed significant LDH release (≥40%) on most of the ONP treatments, we further decided to move ahead with a 3% dosage for other experiments.

#### 3.1.2. Cytotoxicity of BEAS-2B Cells were Exposed to Different Concentrations of Extracts Isolated from Spearmint-Flavored Snus (SKOAL) and ONPs (Zyn)

**Figure 4:**
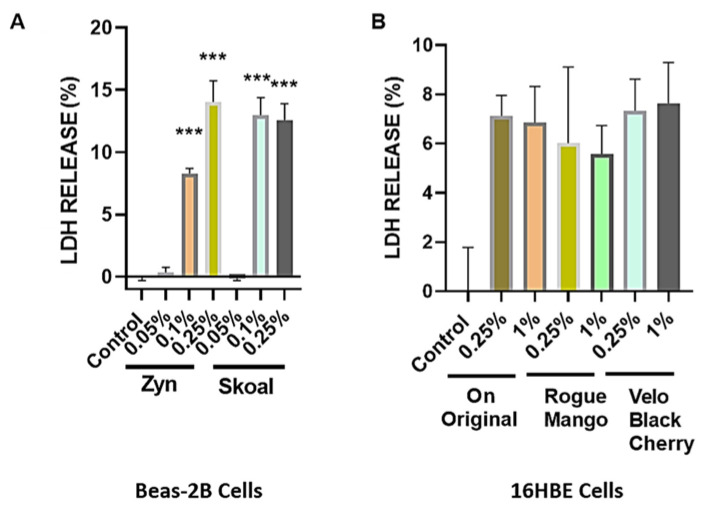
**Differential cytotoxicity due to exposure of flavored oral smokeless nicotine products in bronchial epithelial cells.** (**A**) BEAS-2B cells were seeded and treated with spearmint-flavored oral product extracts from two different brands. Following 24 h exposure, conditioned media was used for the lactate dehydrogenase (LDH) measurements. (**B**) 16HBE cells were seeded and treated with ON! Original, Rogue mango, or Velo black cherry-flavored nicotine pouch extracts. Following 24 h exposure, conditioned media was used LDH measurements. Data represented as means *** *p* < 0.001 compared to control. (Mean ± SEM, *n* = 3.)

BEAS-2B cells were exposed to different concentrations of extracts isolated from spearmint-flavored snus (SKOAL) and ONPs (Zyn). Subsequently, the cytotoxicity of the flavored nicotine ouches was assessed through collecting culture media exposed to 24 h of treatment with a respective extract. The untreated group was considered the control group. Among the tested spearmint products, minimal LDH release was observed at 0.05% concentration, whereas exposure to the 0.1% and 0.25% concentrations exhibited significant levels of LDH release (*p* < 0.01) (Figure 4A)., Both the % LDH release values of BEAS-2B cells exposed to 0.05% Skoal spearmint extract and the 0.05% Zyn spearmint extract did not significantly differ from the corresponding control (*p* > 0.05). Additionally, both the % LDH release values of BEAS- 2B cells exposed to 0.1% (*v*/*v*) Skoal spearmint extract and 0.1% (*v*/*v*) Zyn spearmint extract did significantly differ from the % of LDH released from the corresponding control (*** *p* < 0.001); similar results were seen amongst the 0.25% (*v*/*v*) spearmint-flavored oral nicotine pouch extracts. We further carried out other assays using 0.1% and 0.25% concentrations in light of these observations. Furthermore, 16-HBE cells were individually exposed to 0.25% and 1% extracts of ON! original, Rogue mango, or Velo black cherry ONPs. Differential LDH release was seen among the different flavors of ONPs exposed to 16-HBE cells (Figure 4B).

### 3.2. ROS Production in Human Gingival Epithelium Progenitors (HGEPp) and Human Bronchial Epithelial Cells

**Figure 5:**
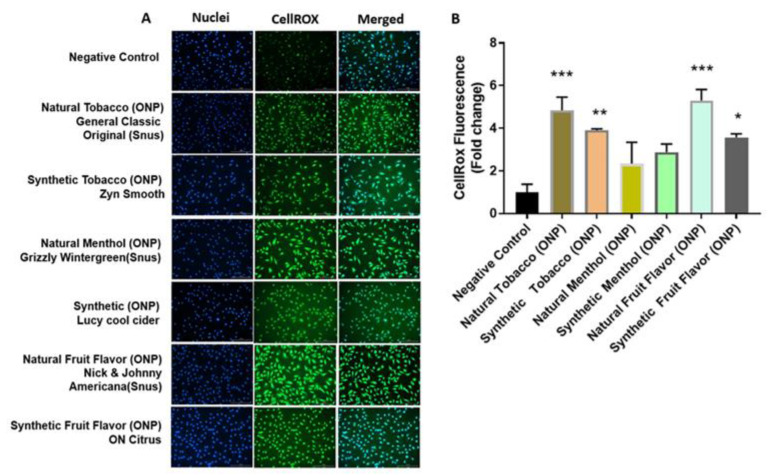
**ROS production in Human gingival epithelium progenitors (HGEPp)**. HGEPp cells were treated at 3% dosage with extracts of oral pouches categorized with natural/synthetic tobacco, menthol, and fruit flavors) belonging to different brands (Snus, Zyn, Grizzly, Lucy, and ON) and incubated for 4 h. Followed by 4 h exposure, cells were exposed to a 5 µM CellRox Green Reagent. Then, were fixed with PFA and nuclei were counterstained with Hoechst 33,342 stain. (**A**) Fluorescence images display the release of ROS and nuclei (blue) in HGEPp. (**B**) Quantitative analysis of fluorescence. Data are represented as mean ± SEM. * *p* < 0.05, ** *p* < 0.01, *** *p* < 0.001. Images were captured by Cytation 5 imaging reader (BioTek) reader and CellROX fluorescent signals were quantified using Image J software (*n* = 3). Scale bar = 200 μm.

The level of ROS production related to the fluorogenic probe was assessed in HGEPp and 16-HBE cells treated with the flavored ONPs of interest. As represented in Figure 5A, HGEPp cells treated with extracts of the flavored ONPs, such as natural tobacco ONP (General original), synthetic tobacco ONP (Zyn smooth), natural menthol ONP (Grizzly wintergreen), synthetic menthol ONP (Lucy cool cider), natural fruit-flavored ONP (Nick & Johnny Americana) citrus and synthetic fruit-flavored ONP (ON! citrus). Natural/synthetic tobacco and fruit-flavored ONP showed significantly higher levels of ROS production compared to the control treatment, whereas natural/synthetic menthol did depict increased cell ROS levels, but the differences were not significant. Figure 5B quantifies the levels of fluorescence in oral cells.

**Figure 6:**
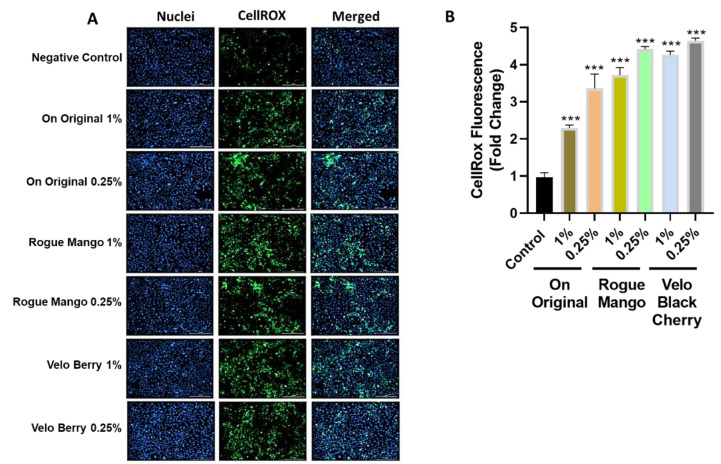
**Production of ROS was measured in 16 HBE cells after treatment with different flavors of nicotine pouches.** 16HBE cells were serum-deprived and were treated with ON! Original, Rogue mango, or Velo black cherry flavored nicotine pouch extracts and incubated for 4 h. Following the incubation oxidative stress measurements were carried out using CellROX Green Reagent. (**A**) Fluorescence images displaying the production of ROS and nuclear morphology (blue) in 16HBE cells treated with different flavors of nicotine pouch extracts at doses of 0.25% and 1%. (**B**) Quantitative analysis of fluorescence in 16 HBE cells. Data are represented as mean ± SEM. *** *p* < 0.001. Images were captured by Cytation 5 imaging reader (BioTek) reader and CellROX fluorescent signals were quantified using Image J software (*n* = 3). Scale bar = 200 μm.

In addition, as shown in Figure 6A, 16-HBE cells treated with extracts of the original, mango, and black cherry-flavored ONPs showed statically significant higher levels of ROS production compared to the control treatment in bronchial cells, Figure 6B counts the levels of fluorescence in lung cells.

### 3.3. Inflammatory Mediator Response Due to Flavoring Nicotine Oral Products in Oral Gingival Epithelium and Bronchial Epithelial Cells

**Figure 7:**
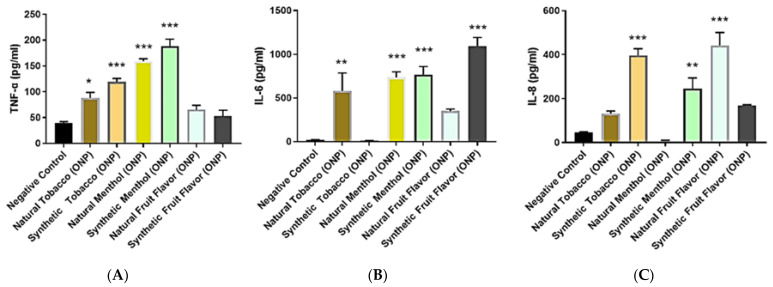
**Inflammatory mediator response by oral nicotine products in oral epithelial cells:** HGEPp cells were treated at oral nicotine pouches extracts categorized with natural/synthetic tobacco, menthol, and fruit flavors) of different brands (Snus, Zyn, Grizzly, Lucy, and ON). Following 24 h exposure, conditioned media was used to perform ELISAs. (**A**) Protein levels of TNF-α determined by ELISA. (**B**) Protein levels of IL-6 determined by ELISA. (**C**) Protein levels of IL-8 determined by ELISA. Data represented as mean ± SEM, * *p* < 0.05, ** *p* < 0.01, *** *p* < 0.001 compared to negative control (untreated) *n* = 3.

HGEPp cells were treated with various natural/synthetic ONPs and Snus, as mentioned above. ONP-mediated pro-inflammatory cytokine release of TNF-α, IL-6, and IL-8 in human oral gingival epithelium cells was assessed via ELISA. Interestingly, enhanced levels of these inflammatory mediators were found as compared to the control. In the case of TNF-α levels, natural/synthetic menthol (Grizzly wintergreen and Lucy cool cider) ONPs as well as natural/synthetic tobacco (General classic original and Zyn smooth) ONP treatment showed significant higher TNF-α levels, whereas natural/synthetic fruit (N&J Americana and ON! citrus) flavor treatments elevated TNF-α non-significantly in oral cells (Figure 7A). Alternatively, IL-6 inflammatory levels were seen to significantly increased in natural/synthetic menthol ONP treatments. Natural tobacco and synthetic fruit also showed increased significant IL-6 levels compared to control cells. However, natural fruit ONPs did show some increase in IL-6 levels but were non-significant. The synthetic tobacco ONP did not affect IL-6 release (Figure 7B). Furthermore, synthetic tobacco, synthetic menthol, and natural fruit ONPs demonstrated significant increased levels of IL-8 levels in oral cells as compared with other flavored ONPs (Figure 7C).

**Figure 8:**
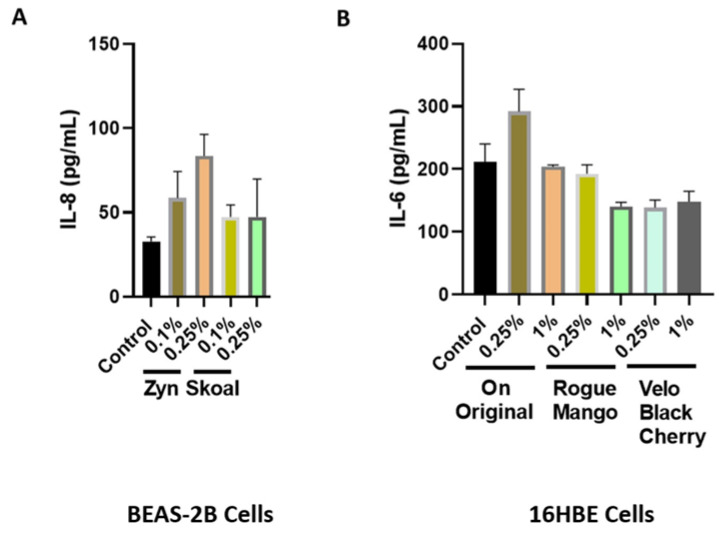
**Inflammatory mediator response due to flavoring nicotine oral products in bronchial epithelial cells**. (**A**) BEAS-2B cells were treated with spearmint-flavored nicotine pouches from Zyn and Skoal brands. Following 24 h exposure, conditioned media was used for IL-8 measurements. (**B**) 16HBE cells were treated with different oral nicotine pouch flavors ON! original, Rogue mango, or Velo black cherry and incubated for 24 h. Following 24 h exposure, conditioned media was used for IL-6 measurement. Results are represented graphically.

Through treatment of BEAS-2B and 16-HBE cells with extracts of flavored ONPs and Snus, ONP-induced inflammatory cytokine responses in bronchial epithelial cells was assessed; this was measure IL-8 and IL-6 concentrations in conditioned media. BEAS-2B cells were treated with spearmint-flavored Snus and ONPs across two concentrations, 0.1%, and 0.25%. Treatment with the spearmint ONP (Zyn) led to no significant change in Il-8 release (Figure 8A). When analyzing IL-6 patterns among 16-HBE cells exposed to extracts of the ON, Rogue, and Velo ONPs, across two concentrations (0.25% and 1.0%), we see that IL-6 levels were not significantly different from those of the control (Figure 8B).

## 4. Discussion

With the prevalence of both ONP and Snus usage and the emerging popularity of flavored ONPs in the U.S., there is a crucial need to better understand the oral and pulmonary health effects of smoke-free nicotine-pouch-based products. Studies have shown that regular use of pouches/smokeless tobacco products induces cytological changes in the oral mucosa [34,35,36], e.g., oral submucosal fibrosis is associated with pulmonary complications in smokeless product users [37]. Additionally, studies have shown regular pouches/smokeless tobacco usage can induce oxidative stress, inflammation, and apoptosis in oral keratinocytes [38,39,40]. Similar studies conducted involving ONPs have so far only used two types of cells in the oral cavity, human oral fibroblasts (HGF) and human gingival fibroblasts (HGF) [29,30]. Additionally, to discern the efficacy of oral-nicotine pouches as a potential nicotine replacement therapy product (NRTP) for those trying to quit vaping, studies that involve analyzing biomarkers of chronic e-cigarette use and e-cigarette- or vaping-associated lung injury (EVALI) using the most popular-brands of ONPs will be crucial. Our study sought to better elucidate how using ONPs may impact periodontal and respiratory cells. To achieve this, we assessed the levels of proinflammatory cytokine release, ROS production, and cytotoxicity in HGEPp and BEAS-2B exposed to extracts of ONPs of various volume concentrations (*v*/*v*%). For oral HGEPp cells, our study utilized six and for BEAS-2B/16-HBE cells we used four of the most widely sold flavored oral nicotine pouch brands in the U.S. (General, Grizzly, Lucy, Nick & Johnny Americana, Skoal, Velo, Rogue, ON!, and Zyn). Additionally, our study involved comparative analyses of inflammation, ROS production, and cytotoxicity among HGEPp and BEAS-2B/16-HBE cells between identically flavored ONPs across multiple extract-volume concentrations. However, no studies utilizing the exposure of oral epithelial cells or 3D EpiOral epithelium to nicotine pouch extracts for analyses of proinflammatory cytokine response, cellular ROS, or cytotoxicity have been conducted. To the best of our knowledge, the present study is the first to report the effect of ONPs on human oral epithelial cells.

Our data suggest that differences in the level of extract-induced cytotoxicity on human gingival epithelium progenitors and bronchial epithelial cells between flavored ONPs are dependent on volume concentrations (*v*/*v*%) of the extract used with the artificial saliva produced. We observed dramatic cytotoxicity differences upon conducting cytotoxicity assays, specifically with the % LDH release levels in the oral gingival epithelium. Cells were treated with extracts of various flavored oral pouches (Tobacco, wintergreen, cool cider, and citrus). Citrus and cool cider demonstrated statically significant differences in cytotoxicity levels across various concentrations (0.25%, 0.5%, 1%, 3%, and 10%), whereas tobacco flavored showed significant cytotoxicity differences only at 3%, and 10% concentrations. Wintergreen flavor did not show significant cytotoxicity changes. Bronchial epithelial cells were exposed to extracts from the spearmint-flavored pouches in this study. We found that differences in cytotoxicity between identical-flavored Snus and ONPs vary depending on the volume concentration of the extract used (0.05, 0.1, and 0.25%). Part of our cytotoxicity data, specifically the % LDH released from BEAS-2B cells treated with Skoal spearmint and Zyn spearmint extracts, align with the finding of another study that utilized cytotoxicity assays using human lung epithelial cells exposed to Snus and oral nicotine pouches by Bishop et al. [29]. Using human gingival fibroblasts (HGF) and human lung epithelial cells (H292) exposed to oral nicotine pouch extracts of various volume concentrations, Bishop et al. had shown that cells exposed to extracts from ONPs showed less cytotoxicity than those exposed to extracts of Snus [30]. Unlike this, the present study reports that the cells exposed to the TFN ONPs extracts showed higher cytotoxicity as compared to those of Snus extracts. Additionally, the flavored ONPs were found to be more determinative and showed higher cytotoxicity on cells comparatively.

To date, the only distinguishing feature between tobacco-derived and synthetic nicotine is its origin. Natural/TDN is obtained from tobacco leaf, and synthetic nicotine is chemically synthesized in laboratories.

Bishop et al., East et al., and our study determined responses of oral and lung cells to ONPs, but our present studies utilized various concentrations on oral cells and evaluated cellular ROS and inflammatory responses upon various oral pouch treatments. The results of the LDH assays conducted in this study differed from that of the findings of a recent similar survey, East et al., which had similarly utilized oral and lung cells exposed to the extracts of Snus and ONPs. East et al.’s findings suggest that regardless of flavor or extract volume concentration, TFN-containing ONPs are less cytotoxic than Snus [30]. Specifically, East et al.’s cytotoxicity assays suggest that LYFT-Revel brand ONPs (now sold under the Velo brand) are significantly less cytotoxic across multiple nicotine concentrations and flavor types than Snus [30]. In our study, the results of the cytotoxicity assays conducted suggest the difference between the tobacco-, citrus-, and cool cider-flavors from the General, Zyn, Lucy, N&J Americana, and ON! extracts in inducing cytotoxic changes in HGEPp cells. Additionally, spearmint-flavored Skoal and Zyn extract in inducing cytotoxic effects in BEAS2-B cells was negligible. However, the difference between our and East et al.’s findings may be attributable to the differences in how our cytotoxicity assays were conducted. Unlike the comparative analyses in cytotoxicity between Snus and ONPs conducted by East et al., those within our study included Snus and ONPs of a single-flavor variety (citrus, cool cider, wintergreen, and spearmint). Additionally, the single type of snus product analyzed in East et al. was the CORESTA Smokeless Tobacco Reference Product (CRP1.1), an unflavored/tobacco-flavored Swedish-style Snus product [30]. Studies have shown that users of ONPs are exposed to lower levels of toxic compounds than users of Snus [4]. Additionally, unlike East et al., which only used TDN- containing ONPs, our study used both TDN and TFN-containing ONPs on oral cells [30]. We further showed dramatic cytotoxicity in oral fibroblast cells by these flavored ONPs (unpublished observations) through ongoing in our laboratory. To corroborate the findings of cytotoxicity, it would be interesting to determine the osmolarity and compare it with pH values, and nicotine content (free or protonated forms) of the pouches/extracts in saliva.

Regarding the release of ROS in response to flavored ONPs from human oral gingival progenitors and human bronchial epithelial cells, our findings indicate a significant differential response with higher levels of CellROX staining in all the treatment groups. HGEPp cells treated with both flavored and unflavored tobacco (original) showed significantly higher ROS production at 3% dose. 16-HBE cells treated with extracts of the original (unflavored tobacco), mango-flavored, and black cherry-flavored ONPs showed slightly higher levels of ROS production when treated with higher doses of extracts by fruity ONPs. Ongoing work with different flavored ONPs (Figure 1A,B) will differentiate the cellular ROS responses including the toxicity on redox homeostasis and mitochondrial function. Differential changes in cytokine release were observed 24 h post ONP treatment. Our findings slightly differed from one study that investigated pro-inflammatory cytokine responses due to exposure to oral pouches/smokeless tobacco [34]. To further explain, Zutsi et al., via quantitative analyses of serum inflammatory cytokine markers such as TNF-α, interleukin IL-6, and interleukin IL-1β, found that among healthy adults, chronic use of oral products is associated with subclinical systemic inflammation [34]. However, in our analysis of pro-inflammatory cytokine levels, we observed elevated expressions of TNFα, IL-6, and IL-8 in oral gingival epithelium. Taking into consideration the present evidence of induced inflammatory changes upon flavored ONPs exposure, ONP exposure could possibly involve different immune cell types, such as neutrophils, macrophages, T-cells, etc., enhancing periodontal and respiratory disease pathogenesis. IL-8 is a neutrophil chemoattractant [41], elevated neutrophil influx might be observed in oral and lung epithelium compartments. Secondly, an increment of IL-6 may activate immune cells participating during acquired immunity via recruiting the effector T-cells [42]. Alternatively, an increase in levels of TNF-α could perhaps modulate responses of macrophages [43] in both oral and lung compartments.

Conversely, we found that regardless of extract-volume concentration (0.1 and 0.25%), BEAS-2B cells exposed to Snus (Skoal spearmint) did not produce significant levels of IL-8. However, this difference between the findings of our study concerning lung epithelial cells and that of Zutsi et al. may be due to the differences in how our inflammatory cytokine analyses were conducted; our study measured the level of a proinflammatory cytokine among primary HGEPp cells and cultured BEAS-2B cells, while Zutsi et al. had assessed proinflammatory cytokine levels using peripheral venous blood [34]. The reasons for this pro-inflammatory response by spearmint may be the presence of flavoring chemicals (e.g., flavor acetals and/or the presence of cooling agents in mint pouches (possibly WS compounds), which requires further chemical analyses.

Consequently, future studies involving analyses of cell responses to extracts from ONPs must include oral fibroblast cells [34,40]. Similarly, a reference smokeless product (CRP1.1) and different nicotine strengths of ONPs (e.g., 3 mg/6 mg) may be used for comparing the results with each other. Our data include cellular responses amongst oral epithelial cells and lung epithelial cells, our findings suggest that there are variations in induced cytotoxicity, generated cellular ROS, and pro-inflammatory cytokine release in oral and bronchial epithelial cells exposed to ONP extracts of various flavors and nicotine strengths. Additionally, these findings indicate the need for further evaluation of ONPs’ role in inducing systemic effects including oral and pulmonary health risks. Further studies are required to study the role of flavored ONPs with different vendors based on the same flavorings at different nicotine strengths which we recently identified based on perceptions [7]. The aforementioned experiment can minimize the role vendor/company plays as a confounding factor in experiments focused on understanding differential flavored ONP-induced oral and pulmonary toxicity based on toxicity assessments among different flavored ONPs for tobacco regulatory science. At the same time, clinical studies are required to assess the toxicity of these emerging flavored ONPs on oral and pulmonary or systemic responses, as shown previously using ENDS flavored products to better understand how systemic changes in inflammation affect the lungs and the pulmonary microvasculature [44,45,46,47,48,49].

Although the focus of this study was on new ONPs that have recently entered the market, other oral tobacco products have been used historically. Prior to the launch of ONPs, earlier studies also reported on the consumption of unburned tobacco products such as Paan and Guthka consumed by people of various regions in Asian countries. These smokeless tobacco products are also consumed by chewing and snuffing ultimately leading to the absorption of nicotine in the lining of the oral cavity [50]. Extensive intake of Paan and Guthka has been reported to cause oral cancer and oral submucosal fibrosis [51]. Evidence also states that prolonged history of chewing Guthka causes post-obstructive pneumonia [52]. The consumption of ONPs can be related to these smokeless products. Use of either of these smokeless tobacco products can damage both the teeth as well as the gum tissue, especially the specific areas of the gum in the mouth causing periodontal gum diseases. Additional studies are required to determine how the toxicological profile of ONPs compare to products used in the past (such as Guthka) or any other oral nicotine products (lozenges, gums, sticks) to ensure they are safer for consumers.

While the study successfully depicted the harmful effects of ONPs on dental and respiratory health using in vitro models, one limitation was the lack of chemical analyses of the ONP products to confirm that the amount of natural and synthetic nicotine delivered was the amount advertised. This could impact our knowledge of these smokeless tobacco products for nicotine delivery and harmful effects on gum microvasculature. Analyzing the chemical characteristics of TDN and TFN ONPs based on flavoring chemicals and other constituents is currently ongoing as well as the investigation into the effect of ONPs on oral and lung fibroblast cells.

**Figure 9:**
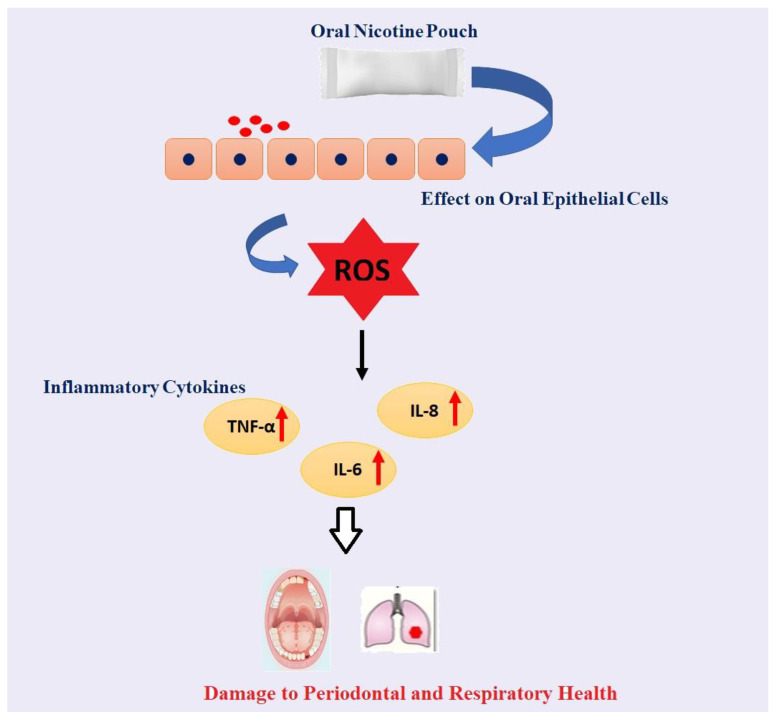
**Schematic demonstration of the effect of Oral Nicotine Pouch on Oral/lung Epithelial Cells**. Chewing of Oral Nicotine Pouch could lead to oral epithelial injury by releasing ROS via activation of inflammatory cytokines such as TNF-α, IL-6, and IL-8 which could further lead to oral and lung-related problems.

Overall, our data showed increased cytotoxicity, the differential release of ROS, cytotoxicity, and cytokine (TNF-α, IL-6, and IL-8) release (Figure 9) at the highest concentration in oral cells and lowest concentration treatments in lung cells at 4–24 h with the most striking response by tobacco-, citrus-, and cool cider-flavored pouches in oral cells both in ROS production and cytokine secretion. Moreover, spearmint showed a better response in lung cells. ONPs containing original tobacco, mango, and black cherry showed higher levels of ROS release in lung cells. These data suggest that both unflavored and flavored ONPs are not safe and likely to cause systemic and local toxicological responses during their chronic usage. Further studies are in progress to determine the oral and pulmonary toxicity of a variety of ONPs flavors using in vitro, ex vivo, and in vivo systems, including human periodontal health. Our study is a part of ongoing efforts to use in vitro, ex vivo, and in vivo systems to understand how the usage of various flavored ONPs may cause both oral and pulmonary toxicity and may impact human periodontal health.

More focused study needs to be addressed with cell signaling pathways using various approaches, which is our next interest to completely dissect the involvement of the probable signaling mechanism highlighting the contribution of the TDN and TFN ONPs influencing the oral and respiratory health.

## Figures and Tables

**Table 1:** Flavored Oral Nicotine pouches treated to Human gingival epithelium progenitors, (HGEPp) cells.

Brand	Product Type	Flavor	Nicotine Concentration	Classification	TDN/TFN
General	Snus	Classic Original	N/A	Tobacco	TDN
ZYN	Pouches	Smooth	6mg	Tobacco	TFN
Grizzly	Pouches	Wintergreen	N/A	Menthol	TDN
Lucy	Pouches	Spearmint	8mg	Menthol	TFN
Nick & Johnny	Snus	Americana	N/A	Fruit	TDN
On!	Pouches	Citrus	8mg	Fruit	TFN

**Table 2:** Flavored Oral Nicotine pouches treated to Bronchial Epithelial cells (Beas2B/16 HBE) cells.

Brand	Product Type	Flavor	Nicotine Concentration	Classification	TDN/TFN
Skoal	Snus	Spearmint	N/A	Spearmint	TDN
On!	Pouches	Original	8mg	Tobacco	TFN
Rogue	Pouches	Mango	6mg	Fruit	TFN
Velo	Pouches	Black Cherry	7mg	Fruit	TFN
ZYN	Pouches	Cool Spearmint	6mg	Fruit	TFN

## Data Availability

We declare that we have provided all the data in the figures. A part of the manuscript was deposited as a preprint on biorxvi.
